# Improving Treatment Adherence with Integrated Patient Management for TB Patients in Morocco

**DOI:** 10.3390/ijerph18199991

**Published:** 2021-09-23

**Authors:** Seup Park, Narae Moon, Byungkwon Oh, Miyeon Park, Kilho Kang, Ilham Sentissi, Sung-Heui Bae

**Affiliations:** 1 Global Care International, Seoul 08377, Korea; park1993able@gmail.com (S.P.); narae710@gmail.com (N.M.); rollzzang@gmail.com (B.O.); my.park@globalcare.or.kr (M.P.); 2 VF Partners, Seoul 06732, Korea; khkang@vfp.co.kr; 3 Chief Public Health Service and Epidemiological Surveillance, Moroccan League Against Tuberculosis (Ligue Marocaine de Lute Contre la Tuberculosis, LMCT), Rabat 10000, Morocco; latdrsrsk@gmail.com; 4 College of Nursing, Graduate Program in System Health Science and Engineering, Ewha Womans University, Seoul 03760, Korea

**Keywords:** tuberculosis, mobile health, community-based treatment, program development, success rate, lost to follow-up rate, Morocco

## Abstract

In Morocco, there are challenges in the management of high-risk tuberculosis (TB) patients, including paper-based management and a shortage of healthcare workers related to TB. Additionally, TB management has not been accounted for in various patient types, which affects treatment adherence. This study aims to examine the delivery model of TB management and the outcomes of an integrated patient management system that uses a patient-centered and community-based approach, along with mobile health technology. A total of 3605 TB patients were enrolled in this program in Morocco’s five prefectures (Rabat, Salé, Kénitra, Khemisset, Skhirat–Témara) from January 2018 to December 2019. Patients were managed based on demographic characteristics, socioeconomic status, areas (rural or urban), health literacy levels, and distance to primary health centers. Our mobile health intervention “smart pillbox” was interposed with high-risk TB patients, along with patient education. The rate of successful treatment was 92.2%, which was higher than the national rate (88%). The “lost to follow-up” rate was 4.1%, which was significantly lower than the existing non-adherence rate of 7.9%. Therefore, integrated patient management for TB patients in Morocco is more effective than the existing conventional programs. This comprehensive approach provides an alternative method for countries with limited resources.

## 1. Introduction

Tuberculosis (TB) is one of the leading causes of death worldwide, with a total 1.4 million deaths in 2019. Globally, the cumulative reduction between 2015 and 2018 was only 6.3%, which is not enough to reach End TB Strategy target of 20% [1]. Pharmacotherapy is an important case management strategy, alongside TB case finding in countries with high TB burden [2]. Treatment adherence interventions are challenging for TB patients [3]. When patients are lost to follow-up, they increase the risk of treatment failure and drug resistance [4]. Lost to follow-up situations often occur to a larger extent in re-treatment cases than in new treatment cases and within the first two months of treatment initiation [5]. Because TB requires long-duration treatment, it is important to monitor TB drug adherence to prevent treatment interruption and the uncontrolled spread of the infectious disease throughout the community [6].

A variety of public health program strategies have been used to improve adherence to TB drugs, including financial incentives and improving coordination and logistics around TB treatment delivery, along with training healthcare providers. Directly observed therapy (DOT) is one of the most commonly used interventions [7]. Digital health technologies, such as video-observed therapy (VOT), short message service (SMS) technology, and electronic pillboxes, are currently used to improve treatment adherence [8,9]. The use of adherence interventions, such as patient education and counseling, incentives, enablers, psychological interventions, reminders and tracers, and digital health technologies, have been shown to improve TB treatment outcomes; moreover, trained healthcare providers and community delivery DOT options also enhance adherence [10]. 

Morocco is a lower-middle-income country. There is a socio-economic difference not only between the rich and the underprivileged but also between rural and urban areas due to rapid urbanization. Even within a city, there are significant differences in health indicators according to different socio-economic statuses [11]. Health priority areas in Morocco have changed rapidly, from communicable diseases to non-communicable diseases. Morocco has been managing tuberculosis by operating conventional Directly Observed Treatment Short course (DOTS), as suggested by the WHO [12]. Morocco practiced a generalized application of TB medication by tuberculosis-related medical staff working in primary health centers. Therefore, in this transition period, there is a great need for changes in TB management, by implementing innovative strategies. It means the conventional DOTS is unlikely to trace TB patients effectively and does not take current social change and individual situations into account. 

The TB incidence rate of Morocco decreased after the introduction of DOTS in 1991; however, the rate remained high at 103 cases per 100,000 population in 2014 [13]. High population density areas even showed increased rates of TB incidence (197 cases per 100,000 population) and increased cases that were lost to follow-up (15%), which represents non-compliance [14]. 

According to the national TB treatment guidelines of Morocco, rifampin, isoniazid, pyrazinamide, and ethambutol are recommended to be taken for 2 months (2RHZE) (6 days out of 7 for 2 months, a total of 56 days) [15,16]. For new smear-positive cases, 4 months of rifampicin and isoniazid (4RH) (6 out of 7 days for 4 months, a total of 112 days), as a category I treatment regimen, are recommended. For patients who had relapsed, streptomycin in combination with RHZE (SRHZE) followed by 1 month of RHZE and 5 months of RHE (2SRHZE/1RHZE/5RHE) are suggested. Prior to self-administered therapy, DOTS is recommended by the Moroccan guidelines for TB patients. The effectiveness of TB treatment is measured both clinically and bacteriologically. 

Therefore, we integrated conventional TB management, DOTS, with a comprehensive patient-centered approach including mHealth, a consideration of the patient’s living area, socio-economic environment, and health literacy level, in order to improve TB medicine adherence and awareness within the community. In addition, our community-based and patient-centered approach using mobile health has revitalized local associations that existed in the country but had not actively functioned to execute programs based on the community. In this article, we describe this program with each component and present its differences from the conventional approach. Finally, we present the early findings to support its effectiveness.

## 2. Materials and Methods

Global Care International (GCI) is an international humanitarian non-governmental organization that is based in Seoul, Korea. We have implemented an enhancement project of TB management capacity using mobile health in Morocco in 2014 in collaboration with the Korea International Cooperation Agency (KOICA) and Morocco Ministry of Health. In addition, there are regional associations in Morocco that can receive support from other Western countries. We partnered with a regional association named La Ligue Marocaine Contre La Tuberculose (LMCT). Since 2018, GCI and LMCT have expanded projects based on a patient-centered approach using mobile health funded by KOICA. 

Our project was conducted in the Rabat-Salé-Kénitra (RSK) region, the second largest region in Morocco, which has a population of about 4,580,866 people [17]. This region was also known to have the second highest TB case notification out of the twelve regions (17.1%) [12]. Among the seven prefectures in Rabat-Salé-Kénitra region, we selected five prefectures: Rabat, Salé, Kénitra, Skhirat-Témara, and Khemisset, which have high tuberculosis incidence rates within the RSK region, and these were further divided into urban and rural areas. In the Témara and Khemisset rural districts, there are smaller numbers of floating populations than those in other areas, with strong community ties. Thus, TB patients can be monitored within the community. These cities were classified as Type 1 cities. Meanwhile, regarding the Salé, Kénitra, and Rabat areas, many rural labor workers live there, but work in other areas. These sectors were then classified as Type 2 cities. 

### 2.1. Treatment Outcomes 

Tuberculosis patients in Morocco obtain medicine from primary health centers every 2 weeks and visit the respiratory disease diagnostic center (Centre de Diagnostic de la Tuberculose et des Maladies Respiratoires, CDTMR) for analysis every 2 months. According to the definition of treatment in Morocco [15], patients become defaulted patients when they do not visit the health center for over 2 weeks and do not receive their medicine. If an absence period is over 2 months or more, and health workers cannot follow up on patient treatment progress or results, then the patient becomes “lost to follow-up.” Therefore, it is necessary to intervene in a patient’s absence with these integrated approaches.

### 2.2. Integrated Patient Management

**Figure 1:**
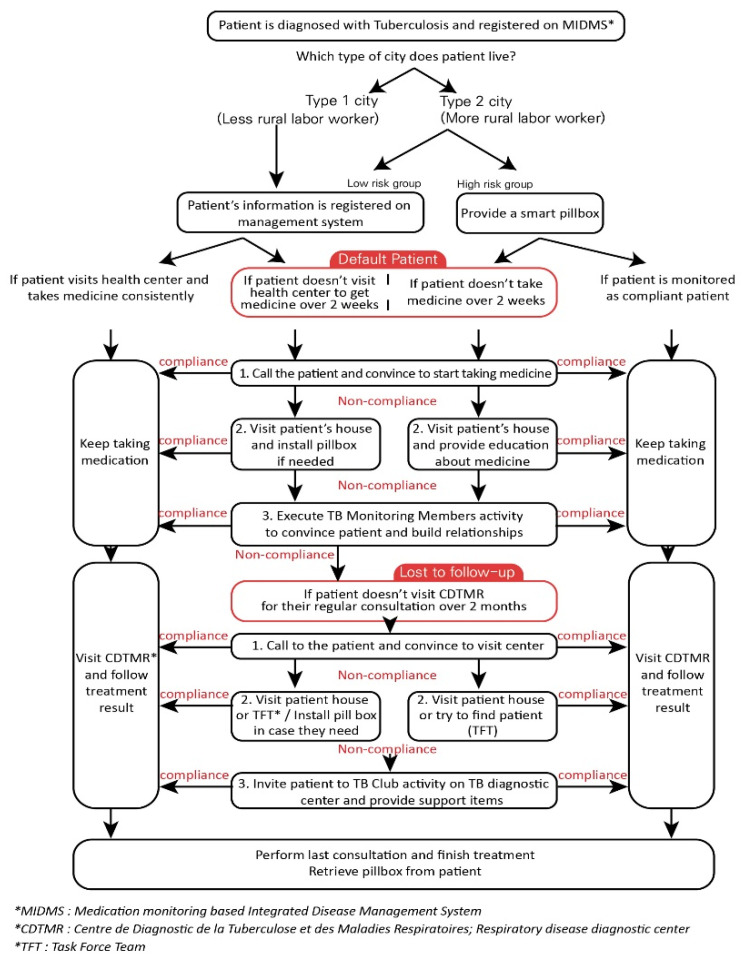
Protocol of the integrated patient management program.

This program was mainly designed by considering a community-based and patient-centered approach, and mobile health was designed and utilized to facilitate the effectiveness of these two factors (community-based and patient-centered). Figure 1 presents detailed information on integrated patient management.

To execute this protocol, mobile health was implemented in accordance with the Moroccan situation. To integrate mobile health into the community context, it was localized and customized in consideration of cultural, social, and regional factors. Morocco has different contexts in urban and rural areas (Types 1 and 2) because there are significant differences in the economic level, health literacy level, care group, residential environment, access to public health centers, male dominance, and gender issues between city and countryside. Therefore, the protocol for dissemination of the smart pillbox and utilization of “Medication monitoring-based Integrated Disease Management System (MIDMS)” by health workers was applied differently. According to this protocol, TB management was provided based on the patient’s situation and context. 

The TB training related to health workers in the primary public health center was performed using mobile phones and the mHealth system in order to cooperate with local associations in the community and provide patient-centered TB care, including high-risk TB patients. The patient tracing system and patient education were also constituted and carried out by community associations as the main axis. We also provided educational programs related to TB medications, considering the health literacy levels of patients and their families.

### 2.3. Program Components 

**Figure 2:**
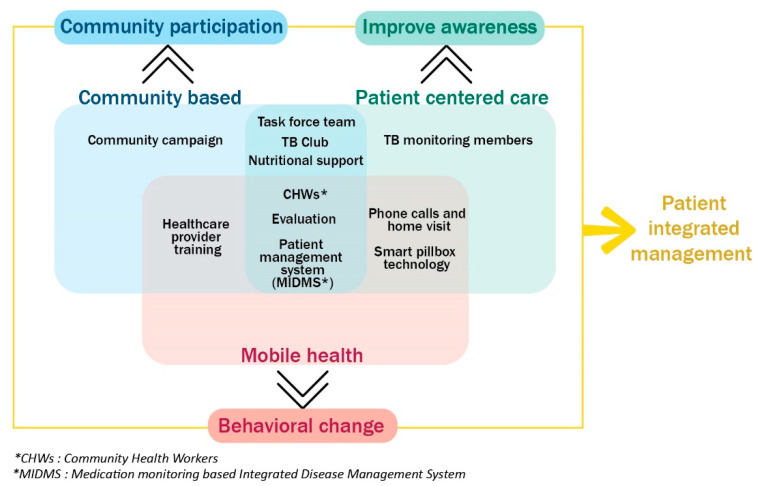
Program conceptual framework.

Figure 2 describes the conceptual framework of our program. We found that mobile health itself can have limited sustainability because of its cost. There are also many challenges in applying mobile health to all beneficiaries in the lower-middle-income countries. Therefore, we tried to address the weakness of mobile health by using a patient-centered approach and considering the patient’s social characteristics within the community.

#### 2.3.1. Community Activity

- Local partner and community health workers

Community health workers (CHWs) are an essential part of this program. GCI coordinated the activities of the CHWs by training and monitoring their work. Since these CHWs are involved in every activity, their understanding of the program is important to be able to manage patients successfully. First, they should know the principle of the mobile health and the process of operating the SP and MIDMS together. GCI and LMCT trained them to manage patients following our TB management program protocol. Finally, the trained CHWs work with TB health providers and take full advantage of mobile health to connect the community with medical institutions.

- Community campaign

TB awareness in the community is a significant factor when TB patients intend to continue and complete their treatment. Through community campaigns, LMCT members can deliver accurate information regarding TB, including its symptoms, contagious periods, and medication. Case-finding activity also progressed during the community campaign. It improves not only public awareness of TB treatment within the community but also helps patient rehabilitation after completing TB treatment. 

#### 2.3.2. Mobile Health

- Mobile health technology

The smart pillbox (SP) is a digital medication monitoring device that notifies patients to take medication at a specific time. It sends the patient’s medication data to the web-based medication monitoring system MIDMS via a subscriber identity module (SIM) card. Through this monitoring system, CHWs or healthcare providers can monitor patients’ medication adherence and manage their treatment using data. It has been proven that SPs are cost-effective and increase treatment success rates [14]. At the same time, MIDMS has an important role in registering patients and their various information, from clinical information to social-demographic information such as the region where they live, financial status, etc.

- Phone calls and home visits

Phone calls and home visits were the most frequent activities among the program components. If a patient did not visit the regional health center to receive TB medicine for at least 2 weeks, community health workers (CHWs) were informed about the patients’ information through the MIDMS or health care providers. Then, CHWs made phone calls to patients and persuaded them to visit the health center and take their TB medicine. However, if patients did not answer the phone call nor follow the given instructions, CHWs visited the patient’s house and tried to meet them in person to give detailed instructions about their TB medication. This activity was continued until patients complied with the TB medicine administration and completed their treatment.

- Health care provider training for mobile health

To enhance TB patients’ management capacity, healthcare provider training was provided to TB nurses, doctors, and administrators. This training was led by the LMCT. Morocco operates the respiratory disease diagnostic center (CDTMR) and primary health centers in each district. In some cases, nurses were responsible for the large number of patients, which makes them not to follow up patients all the time due to heavy workload, and this led to patient dissatisfaction with the healthcare providers’ service. To avoid these situations and increase the quality of care, it is important to provide training programs for TB patient management. Most contents of training were TB patient management using the mobile health system. This training aimed to elicit continuous collaboration with healthcare providers in regional health centers in this program. 

#### 2.3.3. Patient-Centered Activity

- TB Monitoring Members

Even when using phone calls, home visits, and smart pillbox, there are some patients who stop taking TB medicine because of ignorance about tuberculosis and the many difficult circumstances around them. Sometimes patients consider that they are recovered if some of the symptoms disappear or they just give up treatment because of the psychological burden. For patients with lower health literacy, CHWs invited them to have lunch, to build relationships and a close rapport with them. Prior to having lunch with patients, CHWs provided basic information about TB using an animated book for better understanding. Then, they had lunch together with patients and tried to listen to their hardships in life. Through this conversation, CHWs established a rapport with patients and provided a good opportunity to improve patients’ attitudes toward completing TB treatment.

- TB Club

TB Club is a self-help group for patients with TB. CHWs invite both compliant and non-compliant patients. For this activity, patients who have adequate health literacy but do not follow medication well will be invited. When patients join this TB club, they are able to share their experiences, feelings, and challenges they have faced and their way of coping during the treatment process. Healthcare providers also attend the TB club as they provide answers to patients’ questions and look for solutions for patients’ hinderance.

- Task force team

The task force team is an activity searching for lost to follow-up patients. Lost to follow-up refers to a patient who does not visit regular consultation in the CDTMR every 2 months. Most of these patients had difficulty in talking on the phone. Thus, CHWs and health providers collaborate to identify patients with their registered addresses. If CHWs and health providers find the patients or any of their families, they try to persuade these patients to take the medicine again and visit CDTMR to start their treatment process again.

- Nutritional Support

Throughout the medication period, proper nutritional care is needed for patients with TB. However, many TB patients in Morocco are underprivileged and unable to purchase sufficient nutritional supplements. Therefore, nutrition support bags consist of essential ingredients, such as flour, rice, cooking oil, milk, canned tuna, etc. were supplied. When CHWs visit a patient’s house, they check their financial status. Then, the CHWs make the decision to provide a nutritional support bag during their treatment.

- Evaluation

Each time we ended patient sensibilization activities, such as TB monitoring members and healthcare provider training, we conducted a knowledge survey and satisfaction survey. A mobile survey tool (Google Forms) was used, and CHWs conducted evaluations using a mobile phone.

### 2.4. Data Analysis 

To analyze the effectiveness of this program, we reviewed the patients enrolled in the MIDMS. We identified patients’ information regarding whether they had recurrence, treatment results, and whether they used a smart pillbox. All patients’ identification data was removed, and data analysis was performed with the review and consent exemption of the approval of the Moroccan Ministry of Health.

All data analysis was conducted using SAS 9.1 (SAS Institute Inc., Cary, NC, USA). Descriptive statistics were used to examine study variables and outcomes. We used the Chi-squared test and the independent *t*-test to compare the study variables and outcomes in the Type 1 and Type 2 areas.

## 3. Results

**Figure 3:**
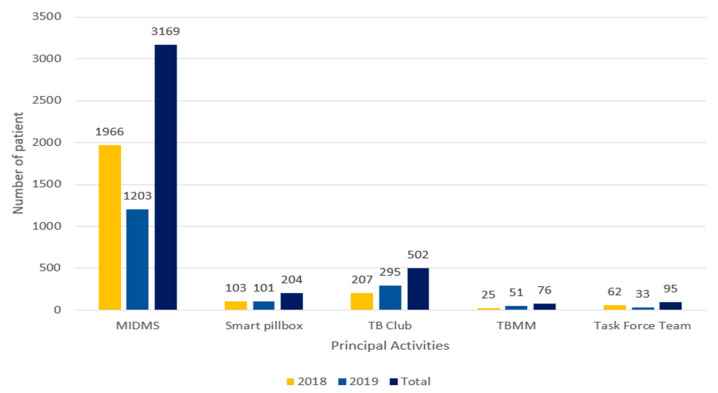
Project components related to treatment success.

In this study, we analyzed a total of 3438 patients enrolled from 2018 to 2019 in integrated patient management. Figure 3 presents the number of patients who participated in project components, including MIDMS, smart pillbox, TB club, TBMM, and Task Force Team. These project components are considered to affect treatment success rates of TB patients. In total, 3169 patients enrolled in MIDMS and 204 patients used smart pillbox. 

As seen in Table 1, 2138 (62.2%) were male, and the average age was 37.5 (standard deviation 51.1). Analysis was also conducted by dividing the Type 1 and Type 2 areas according to the implemented protocol. A total of 3260 (94.8%) new cases with no history of tuberculosis and 170 cases of relapsed cases were also noted. The rate of relapse was 5.4% in the Type 2 area, slightly higher than the 3.3% in the Type 1 area. Eight patients with multidrug-resistant (MDR) TB were also enrolled in the Type 2 area. The treatment type was significantly different by area.

The treatment results of the patients can be classified into six types: cure, treatment completed, treatment failure, death, lost to follow-up, and not evaluated. If the treatment success rate is considered as the number of patients who are cured or have completed the treatment, we have a success rate of 91.7% in the Type 1 area, 92.4% in the Type 2 area, and 92.2% overall as shown in Table 1. Both the Type 1 and Type 2 areas show high treatment success rates when the current program is executed, and it is higher than the 88% of Morocco’s existing treatment success rate [12].

Table 2 presents detailed information on the baseline characteristics and treatment outcomes of TB patients by gender. There is no difference in the mean age by gender. Compared to that of female patients (3.8%), a greater proportion of male patients (5.7%) included relapsed cases. We found that the treatment success rate in female and male patients was 93.3% and 91.5%, respectively. 

## 4. Discussion

This paper attempted to compare the effectiveness and differences between the conventional tuberculosis management approach in Morocco and the patient integrated management developed by GCI. We noted an over 90% treatment success rate in both Type 1 and Type 2 areas. A total of 1248 (96.0%) new cases in female patients were found, which was a little higher than that in male patients. A greater proportion of male patients had relapsed cases compared to that of female patients. Overall, treatment success rates were more than 90%.

Top-down TB treatment approach in Morocco reduced the likelihood of treatment success rate among patients with TB and had the difficulty in managing non-compliant patients. Such an approach is more reliant on healthcare workers in health facilities [18]. It is important to consider that these people in the workforce have many other responsibilities on top of following up TB patients to complete their TB medication and ensure their adherence [10]. In particular, more treatment defaults are bound to appear among the marginalized population in Morocco, such as women, mentally ill, and urban poor due to the lack of accessibility to health facilities [16,19]. In the previous study, the treatment success rate of TB patients in our project area was 79.5% with conventional DOTS [14].

Therefore, as an alternative to conventional DOTS, GCI attempted to manage treatment adherence through integration within the community by categorizing individual characteristics according to social, cultural, and environmental factors. GCI also applied social changes such as Morocco’s urbanization, changes in individual lifestyles, and individualization into a community-based patient-centered approach with innovative technologies. 

Table 3 presents a comparison between conventional DOTS [19,20] and integration patient management in our project. Previous studies [19,20] showed the conventional DOTS in Morocco. Patients had a lack of knowledge and understanding, and healthcare providers experienced a lack of financial and staff resources to identify patients who had defaulted treatment [19]. Although integrated patient management has some significant differences from conventional DOTS, it was conducted in addition to the existing method through the convention with Morocco’s Ministry of Health rather than running two separate programs. As a result, we have both the control arm of the Ministry of Health and a participatory community engagement arm.

In addition to the integration of patient management, several technologies have been integrated into the program. During the previous stage of the program (2014–2018), smart pillbox, telephone, and TB patient’s management platform, MIDMS, were used by most beneficiaries. However, during this stage, we found that the treatment success rate could be increased with only MIDMS and telephone communication depending on the type of city, patient’s living condition, and educational level. The patient-centered approach could be applied via the analysis of individual medication adherence levels through the use of MIDMS at health facilities. Therefore, the smart pillbox was used for patients who had lower adherence levels in the urban context or were difficult to follow up in the region with limited number of health professionals at the community centers. Nonetheless, the smart pillbox continued to serve as a point of contact among patients, community health workers, and health providers. Various patient education programs were provided according to the levels of patients’ health literacy on tuberculosis and willingness to take the medicine regularly as a part of the patient-centered approach. Lastly, treatment success rate and default rate were improved through multifaceted social support activities such as financial and nutritional support items, psychosocial support, counseling, consulting, and patient welfare, among others.

We developed a protocol for providing education, smart pillbox, and psychological support according to characteristics of residential area, socioeconomic status of each patient with TB, level of health literacy, and the presence of caregivers. 

In fact, the number of patients with MIDMS, smart pillbox, TB club, TBMM, and Task Force Team (MIDMS), which affected the treatment success and re-treatment of patients, showed that the mobile health element with MIDMS and smart pillbox was applied to the largest number of patients (Figure 3). However, patient-centered activities, including patient education, can also be seen as having a positive impact on patients’ treatment outcomes, even if the input is not high. A detailed analysis is expected to be done in the future program’s qualitative research. Therefore the mobile health component, when combined with community-based approach and patient-centered approach, showed synergy to produce effective results. Higher treatment success rates were found not only in patients who participated in our project but also in the total number of patients in the project area. For example, the national tuberculosis treatment success rate was 88%, which is higher than that in Salé (76%) [12,21]. This national rate did not increase between 2016 and 2019. Many interventions are put into effect for tuberculosis management every year in Morocco; however, it is hard to determine significant improvements in the treatment success rate. On the other hand, the total treatment success rate increased from 76% to 83% between 2016 to 2019 in Salé [21]. Salé is one of our project areas, and we have been working with the smart pillbox since 2015; thus, this result shows the effectiveness of our project on complete tuberculosis management in Salé.

Morocco is a region of the Francophonie, where health policy controls TB through local associations. In cooperation with the local tuberculosis association, we can not only manage TB patients by mobile health, but also implement health providers’ training programs and the TB campaign in the community. 

As the program progressed, we discovered some challenges. First, the method has a higher cost compared to the conventional method, partly because of the patient management carried out through various interventions such as the use of IT-related technology and nutritional support. However, it is more cost-effective than the conventional method according to the unpublished KOICA monitoring and evaluation team’s study. Unstable internet connectivity issues for devices pose another challenge. New interventions, specifically IoT-related technology, are dependent on the internet connection for sending and retrieving data. We have various responses to the challenge of the internet, such as regularly using WIFI dongles in regions with weak internet connectivity in order to collect the data of medication.

There is considerable research in other countries that emphasizes novel approaches toward TB patient management. In South Africa, Brust and colleagues implemented an integrated home-based MDR–TB–HIV treatment program and tried to decentralized care for patients [22]. There is another research in Ghana, and this study sought additional activities for the further improvement of patient management [23]. They found low level community contribution at their research site, and it was linked to the low treatment success rate. In Ethiopia, Getahun, and Nkosi [24] also identified the limitations of conventional DOTS and the need for a patient-centered approach. Another study in Ethiopia implemented a trial with an electronic pillbox, and the authors verified the impact of mobile health on treatment adherence [25]. However, all these studies utilized one of the components among community-based, patient-centered, and mobile-health interventions separately. In contrast, our project has a unique contribution in that it integrated all of these components and produced higher treatment success rates among TB patients in Morocco.

In future studies, further examination should be performed using empirical data to verify the effectiveness of our project. Based on the concept of the project, we also recommend that this program be applied to other countries of Francophonie that have different socioeconomic and geographical environments.

## 5. Conclusions

In conclusion, integrated patient management resulted in higher treatment success rates than existing programs in Morocco. This comprehensive approach provides an opportunity to overcome hurdles in TB patient management. However, there are still challenges in the human resources and technology infrastructure that are required to execute this program. Therefore, continuous attention from the Morocco Ministry of Health and Global Society is essential to expand this program nationwide and be carried out in other Francophonie countries.

## Figures and Tables

**Table 1:** Baseline characteristics and treatment outcomes of TB patients (January 2018–December 2019).

Variables	TB Patients in Both Areas (n = 3438)	TB Patients in a Type 1 Area (n = 793)	TB Patients in a Type 2 Area (n = 2645)	*X*^2^ or *t* (*p*-Value)
	n (%)	n (%)	n (%)	
Gender				
Male	2138 (62.2%)	487 (61.4%)	1651 (62.4%)	0.26
Female	1300 (37.8%)	306 (38.6%)	994 (37.6%)	(0.608)
Age				
Mean (SD)	36.40 (18.63)	37.06 (18.82)	36.21 (18.58)	1.12 (0.261)
Treatment type				
New case	3260 (94.9%)	767 (96.7%)	2493 (94.3%)	8.58
Relapsed case	170 (4.9%)	26 (3.3%)	144 (5.4%)	(0.014)
MDR case	8 (0.2%)	0 (0.0%)	8 (0.3%)	
Treatment outcome				
Cured	1316 (38.3%)	251 (31.7%)	1065 (40.3%)	29.23
Treatment completed	1853 (53.9%)	476 (60.0%)	1377 (52.1%)	(<0.0001)
Treatment failed	29 (0.8%)	1 (0.1%)	28 (1.1%)	
Died	62 (1.8%)	13 (1.6%)	49 (1.9%)	
Lost to follow-up	123 (3.6%)	36 (4.5%)	87 (3.3%)	
Not evaluated	55 (1.6%)	16 (2.0%)	39 (1.5%)	
Treatment success				
Yes	3169 (92.2%)	727 (91.7%)	2442 (92.3%)	0.36
No	269 (7.8%)	66 (8.3%)	203 (7.7%)	(0.551)
Mobile health outcome				
Patients with smart pillbox				
Yes	206 (6.0%)	64 (8.1%)	142 (5.4%)	7.91
No	3232 (94.0%)	729 (91.9%)	2503 (94.6%)	(0.005)

Note: TB, tuberculosis; SD, standard deviation; MDR, multidrug-resistant tuberculosis.

**Table 2:** Baseline characteristics and treatment outcomes of TB patients by gender (2018.1–2019.12).

Variables	Male (n = 2138)	Female (n = 1300)	*X*^2^ or *t* (*p*-Value)
Age			
Mean (SD)	36.75 (18.08)	35.85 (19.51)	–1.37 (0.171)
Treatment type			
New case	2012 (94.1%)	1248 (96.0%)	6.15 (0.046)
Relapsed case	121 (5.7%)	49 (3.8%)	
MDR case	3 (0.2%)	5 (0.2%)	
Treatment outcome			
Cured	886 (41.4%)	430 (33.1%)	40.70 (<0.0001)
Treatment completed	1070 (50.0%)	783 (60.2%)	
Treatment failed	19 (0.9%)	10 (0.8%)	
Died	39 (1.8%)	23 (1.8%)	
Lost to follow-up	93 (4.4%)	30 (2.3%)	
Not evaluated	31 (1.5%)	24 (1.8%)	
Treatment success			
Yes	1956 (91.5%)	1213 (93.3%)	3.71 (0.054)
No	182 (8.5%)	87 (6.7%)	
Mobile health outcome			
Patients with smart pillbox			
Yes	130 (6.1%)	76 (5.9%)	0.079 (0.779)
No	2008 (93.9%)	1224 (94.1%)	

Note: TB, tuberculosis; SD, standard deviation; MDR, multidrug-resistant tuberculosis.

**Table 3:** Comparison of conventional DOTS and integrated patient management.

Conventional DOTS	Integrated Patient Management
**Patient-centered aspect**	
Challenge to trace lost-to-follow up patient	Facilitating the management of non-compliant patients effectively
Undifferentiated approach to all patients	Diverse approach based on type of city, patient’s socio-economic status, individual conditions (health literacy, family situation)
**m-health aspect**	
Delayed data collection and decision making	Real-time monitoring and surveillance
Risk of loss of data	Safe data storage
High burden of workload on health providers	Reduced workload of health providers
**Community-based aspect**	
Centralized care, low accessibility	Close care within the community, high accessibility
Unilateral communication from health providers	Promoting bilateral communication between patient and health providers
Less awareness within the community	Community participation
Limited task of health providers	Health provider’s greater commitment
Low cost	High cost

## Data Availability

Data sharing not applicable.
